# Effects of Polyurethane Small-Sized Microplastics in the Chironomid, *Chironomus riparius*: Responses at Organismal and Sub-Organismal Levels

**DOI:** 10.3390/ijerph192315610

**Published:** 2022-11-24

**Authors:** Sara A. M. Silva, Andreia C. M. Rodrigues, Teresa Rocha-Santos, Ana L. Patrício Silva, Carlos Gravato

**Affiliations:** 1 Department of Biology, University of Aveiro, 3810-193 Aveiro, Portugal; 2 Centre for Environmental and Marine Studies (CESAM) and Department of Biology, University of Aveiro, 3810-193 Aveiro, Portugal; 3 Centre for Environmental and Marine Studies (CESAM) and Department of Chemistry, University of Aveiro, 3810-193 Aveiro, Portugal; 4 Faculty of Sciences, CESAM, University of Lisbon, Campos Grande, 1749-016 Lisbon, Portugal

**Keywords:** benthic macroinvertebrates, life history traits, behaviour, biochemical responses, small plastic particles

## Abstract

Freshwater provides valuable services and functions to humankind. However, macroinvertebrates that underpin the delivery of many of those ecosystem services and functions are under an additional threat caused by microplastic pollution. Chironomids are one of the most abundant groups of macroinvertebrates in these environments and the most sensitive to microplastics. This investigation addressed the effects of polyurethane (PU-MPs; 7.0–9.0 µm) on the chironomid *Chironomus riparius* at the organism and sub-organism levels. For this purpose, two assays were carried out: (i) addressing the effects of PU-MPs on *C. riparius* partial life cycle traits (larval size and emergence parameters) in a 28 d assay considering concentrations up to 750 mg/Kg, and (ii) larvae behaviour (locomotion) as well as the biochemical responses (oxidative damage, aerobic energy production, and energy reserves) in a 10 d assay considering an environmentally relevant concentration with no observed effects on *C. riparius* previous life history traits (no observed effect concentration; NOEC = (375 mg/kg). Exposure to PU-MPs did not affect *C. riparius* larval length nor cumulative and time to emergence. Conversely, when exposed to an environmentally relevant concentration for 10 days, contaminated larvae were revealed to be lighter (but not smaller nor less nutritionally affected in terms of energy reserves) and more active when foraging, which was reflected in the activation of their aerobic metabolism when assessing the electron transport chain as a proxy. Notwithstanding, PU-MPs did not originate observable energy costs, either on protein, lipid, or sugar contents on contaminated larvae, which may justify the absence of effects on larval growth and emergence. Therefore, the increased production of energy used for the locomotion and functioning of larvae was at the expense of the fraction of energy that should have been allocated for the weight of the individuals. A long-term exposure involving a multigenerational assessment would bring intel on the potential (cumulative) sub-lethal effects of PU-MPs on *C. riparius* fitness.

## 1. Introduction

Freshwater ecosystems provide essential direct and indirect contributions to human well-being (also known as ecosystem services), such as the provision of purified water, food source, nutrient mobilisation, flood alleviation, and the opportunity for recreational activities [1]. However, freshwater biodiversity, particularly of macroinvertebrates that underpin the delivery of many of those ecosystem services, is under an additional threat caused by plastic pollution. In fact, freshwaters are among the major collectors and pathways of plastic debris to the sea. Here, plastic debris can reach the order of grams per litre in surface waters or grams/thousands of particles per kilogram in sediments [2,3,4].

Chironomids are often the most abundant group of macroinvertebrates in the number of species and individuals encountered in freshwater environments, particularly in benthos [5]. Concerningly, they are also one of the most sensitive groups to the presence of microplastics in sediments, revealing the high ingestion rates, bioaccumulation, and adverse effects in laboratory conditions, when considering life history traits and biochemical/physiological response mechanisms [6,7,8,9,10,11,12] (Table 1). In addition, the decrease in such organisms (along with other detritivores and shredders) in a mesocosms experiment with contaminated sediments with microplastics (Polyethylene- PE, concentrations from 0.1 to 10 g/kg) significantly (and negatively) affected freshwater macroinvertebrate community (abundance, structure, and composition), although no significant effects on organic matter decomposition nor primary production were denoted potentially due to the short test duration (e.g., [13]).

Ecotoxicological effects on chironomids seem to be highly dependent on microplastic size, shape, and polymer type (Table 1) (e.g., [14,15]). Thus, considering their ecological role in freshwater ecosystems (e.g., nutrient mobilisation and position in freshwater food webs, among others [16]), it is essential to address the potential effects of other polymers of different sizes and shapes in this group of organisms. For a deeper understanding of the extent of the potential ecotoxicological effects of new polymers, it is also important to test environmentally relevant concentrations of microplastics and provide data and knowledge, considering their effects at different levels of biological organisation, i.e., from cell to population.

The most tested polymers belong to the thermoplastics (e.g., PE, polyvinyl chloride-PVC, polyethylene terephthalate-PET, polyamide-PA, see Table 1), and are mainly characterised by a lighter structure with Van der Waals interactions [17]. Conversely, little is known considering thermosetting plastics, such as polyurethane-PU, a group characterised by crosslinking with strong chemical molecular bonds, higher molecular weights, and stronger structures [17]. Studies comparing the effect of both thermoset (PU) and thermoplastics (PE, polypropylene-PP, PA) reported higher toxicity in the former, in lotus plants (*Nelumbo nucifera* [18]), and bacteria (*Aliivibiro fisheri* [19]). To increase the knowledge of the potential effects of thermoset plastics in chironomids, this study addressed the effects of small-sized polyurethane MPs (PU-MPs) on *Chironomus riparius* at different levels of biological organisation. Firstly, we addressed the effects of different concentrations of PU-MPs on chironomids’ life history traits (survival, larval growth, and imagoes emergence) on a 28 d assay. Afterward, and considering a no observed effect concentration (NOEC) but one that is environmentally relevant, obtained from the life history assessment assay, the behavioural (locomotion) and biochemical responses (oxidative damage, aerobic energy production, and energy reserves) of the fourth instar larvae were characterised after 10 days of exposure.

**Table 1:** Summary of the impact of microplastics on Chironomids.

Chironomid Species	Polymer Type, Size, and Concentrations Tested	Main results	
*Chironomus riparius*	Polyethylene (PE, 32 to 64 μm; [MP] up to 20 g/kg)	Reduced larval growth and delayed imagoes emergence	[6]
		Induced oxidative damage and reduced aerobic energy production	[9]
		Increased basal phenoloxidase activity	[20]
	Polyethylene terephthalate (PET, 14 μm; [MP] up to 5 × 10^5^ items/kg)	Emergence, weight, or head capsule lengths were not affected	[14]
	Polystyrene and tire rubber (PS and TR, 38 and 82 μm, respectively; [MP] up to 10 mg/L)	Caused cellular stress	[8]
		Some alterations in gene expression	
	Polyvinyl chloride (PVC, <50 μm; [MP] up to 20 g/kg)	Reduced the emergence and weight	[12]
	PET, PS, PVC, and polyamide (PA) (10–400 μm as mixture; 8–80 g/m^2^ sediment)	Increased body mass, larval body length, mouthparts deformities, and female imagoes wind deformities	[11]
*Chironomus tepperi*	PE (1 to 126 μm, 500 items/L)	Affected survival, growth, and emergence	[7]

## 2. Materials and Methods

### 2.1. Culture Conditions of Chironomus riparius 

*Chironomus riparius* were cultivated and maintained under controlled conditions at the Technological Laboratories building, Chemistry Department, University of Aveiro. Cultures were maintained in reconstituted hard water [21] according to the guidelines [22]. A stock of 20 L of this medium (also used in the tests) consisted of 17 L of ultrapure water (which comes from the action of a distiller and a purifier with filters on the mains water), 200 mL of solution A (0.16 g/L KCl), 200 mL of solution B (4.91 g/L Mg SO_4_.7H_2_O), 200 mL of solution C (3.84 g/L NaHCO_3_), 1.5 L of solution D (2.4 g/1.5 L CaSO_4_), and 1 mL of vitamins (1.5 mg of thiamine HCl + 0.02 mg of cyanocobalamin + 0.15 mg of biotin in 1 mL of ultrapure water). The solution pH must be set between 7.3 and 7.5 with 5N HCl. 

Organisms were reared in glass aquaria (30 × 20 cm) at 20 ± 1 °C under a 16:8 h light–dark photoperiod. Larvae were grown in a layer (~3 cm) of previously burnt inorganic sediment (500 °C for 4 h) and the American Society for Testing Materials (ASTM) hard water in a 1:4 ratio (ASTM, 1980) with continuum aeration. Adults were confined within an acrylic cage covered with mesh netting. Feeding consisted of macerated commercial food fish TetraMin^®^ (Tetrawerke, Melle, Germany) (nutritious food for aquarium animals) macerate that was provided three days a week (ad libitum).

### 2.2. Polyurethane Microplastics Used in the Experiment

The polymer chosen for this study was Polyurethane (PU), an odourless white powder composed of aliphatic PU with a density of 1.05 (g/cc) and an average particle size between 7.0 and 9.0 μm, kindly provided by a local enterprise that prefers to remain anonymous. Such polymer is of interest due to its intensive use in different sectors, from medicine to the building sector, particularly in building insulation, to achieve higher sustainability [23].

### 2.3. A 28-Day Exposure and Evaluated Endpoints

The 28 d assay followed the international guidelines for sediment–water chironomid life cycle toxicity tests [22] with slight adaptations. Briefly, four *C. riparius* egg masses were isolated in glass flasks containing ASTM until hatching. Then, first instar (<48 h post-hatching) larvae were used in chronic 28-day life cycle assays. The tested concentrations were 93.5, 187.5, 375, and 750 mg of PU-MPs/Kg sediment. This concentration range was chosen to include the same order of magnitude of concentrations used on previous tests for comparison purposes (e.g., [6,15]) and concentrations expected in the field for small-sized MPs [24]. Each PU-MP condition and a control (uncontaminated sediment) consisted of ten replicates with 50 g of contaminated sediment (except for control treatment where uncontaminated sediment was used) that were gently filled with 150 mL of ASTM hard water. Such vials were allowed to equilibrate for 3 days prior to adding five larvae to each vial. After 10 days, larvae from five control replicates and each PU-MP concentration were used to measure the total body length after sacrificing them with 70% ethanol. The other five replicates of five organisms were used for analysing the emergence of imagoes (adult insects) until the end of the test. Such imagoes were trapped in a plastic container inserted on top of the glass test vial. Daily emerged imagoes were collected and counted. 

Throughout the experiment, larvae were fed three days a week (0.25 mg of macerated TetraMin per organism per day), and the test conditions were the same as described for culturing. The life cycle experiment lasted 28 days until the last adult emerged. From this assay, a no observed effect concentration (NOEC, 375 mg/kg) was chosen for the short-term exposure assay (10 days, following section), to reduce animal experimentations to the minimum [25] and assess behavioural, physiological, and biochemical endpoints.

### 2.4. A 10-Day Exposure and Evaluated Endpoints

Similar to in the previous assay, four *C. riparius* egg masses were isolated in glass flasks containing ASTM until hatching. The larvae (<48 h) were equally transferred to three glass aquaria (30 × 20 cm) containing 800 g of sediment with 2 L hard water (control condition) or three glass aquaria containing 800 g spiked sediment with 2 L hard water (375 mg PU-MP/kg—a NOEC; equilibrated for 3 days before larvae addition), for a period of 10 days at 20 ± 1 °C under a 16:8 h light–dark photoperiod.

Again, the addition of ASTM was performed by gently pouring the ASTM hard water to minimise the resuspension of PU-MPs mixed in the sediment. The test aquaria were then covered with lids and allowed to equilibrate for three days (a time consisting of the hatching of isolated egg masses). After the hatching of the masses, larvae were equally divided per aquarium, thus initiating the exposure of the larvae. Each aquarium was provided with aeration. Larvae were fed 3 days a week with macerated TetraMin^®^ diluted in ultra-pure water, as described in the previous section. 

After exposure, fifteen random larvae were collected from control and PU-MP treatment, measured to confirm larval body length (also addressed in the first test), and sacrificed in 70% ethanol to verify larvae PU-MP uptake. From each aquarium (the control or PU-MP treatment), four replicas of five larvae were also collected and transferred to 250 mL glass vials containing 40 mL of ASTM hard water to evaluate larvae behaviour (locomotion). Finally, another five replicates of fifteen larvae were collected from each aquarium (the control or PU-MP treatment), gently rinsed, dried on filter paper, put into 2 mL microtubes, weighted (fresh weight, FW), snap-frozen in liquid nitrogen, and stored at -80 ˚C to further assess oxidative damage (via LPO), energy reserves (lipids, proteins, and carbohydrates) and energy consumption (via ETS). 

### 2.5. Total Body Length Measurement

Larvae body length was measured from the base of the head capsule to the anus using a USB microscope and a micrometre calibration ruler. This procedure was performed in larvae (10 days old and fourth instar) from both bioassays.

### 2.6. PU-MP Presence in the Larval Digestive Tract

Microplastic extraction from *C. riparius* tissues, and their quantification followed previous investigations [6,26]. Briefly, larvae were gently dried on filter paper and transferred to glass tubes and 2 mL of nitric acid (HNO_3_; 65%) was added for 3 h at 60 °C. To complete the digestion process, the samples were cooled to room temperature, and a volume of 2 mL of hydrogen peroxide (H_2_O_2_; 35%) was added for 24 h. Once the samples were cleared of visible oxygen bubbles, replicas were diluted using Milli-Q water in a 1:10 ratio [6,20]. Solutions resulting from digestion were filtered through a glass vacuum system onto black polycarbonate filters (PCTE, 0.2 μm pore size, 47 mm ∅, ref. 7063-4702, GE Healthcare Whatman^TM^), stained with Nile Red (Sigma Aldrich, USA; 100 μg/mL in absolute ethanol) for 2 min, and washed abundantly with ultrapure water. Afterward, filters containing PU-MPs were observed and photographed under a microscope (Olympus BX41; objective 10×) copulated with a Canon EOS 1200D camera. This entire procedure took place in a fume hood covering samples and filters to avoid potential airborne contamination by MPs. Additionally, all the equipment and test vials were acid-washed and thoroughly rinsed with Milli-Q water.

### 2.7. Larvae Behaviour (Locomotion) Assessment

Larvae were filmed for 2 min (exact time), and the number of curling and uncurling (the typical behaviour of chironomid larvae while foraging) was recorded. Data were presented as the number of curling/min per treatment.

### 2.8. Biochemical Parameters Assessment: Oxidative Damage (via LPO), Energy Reserves (Lipids, Proteins, and Carbohydrates), and Energy Consumption (via ETS)

Samples were defrosted on ice and homogenised in 1600 μL of ultra-pure water using the ultrasound Fisherbrand™ Model 120 Sonic Dismembrator. From such homogenate, three aliquots of 300 µL were collected from each sample: one for analysis of lipid content, one for sugars and protein content, and the third one to evaluate ETS activity. For LPO determination, a 200 µL aliquot was collected from the remaining homogenate, to which it was added 4 µL of 4% BHT (butylated hydroxytoluene). Measurement of lipid peroxidation was conducted according to Campos et al. [27], measuring the levels of thiobarbituric acid-reactive substances (TBARS) at 535 nm [28]. Results are expressed in nmol TBARS/g organism.

Available energy (energy reserves) and aerobic energy production (ETS) were determined following the method by De Coen and Janssen [29], following the adaptations by Campos et al. [27] and Rodrigues et al. [30]. For total lipid content, one of the groups of 300 µL aliquots were centrifuged (1000× *g* or 3500 rpm for 5 min) after the addition of 500 μL of chloroform (≥99.8%) and 500 μL of methanol (≥99.8%) and the organic phase were then transferred to a glass tube, acidified using 500 μL sulphuric acid (H_2_SO_4_) and incubated at 200 °C for 15 min. After cooling, 1500 μL of ultra-pure water was added to each tube. The absorbance of samples and tripalmitin, for the calibration curve, was measured at 375 nm. 

Carbohydrate and total protein contents were determined after adding 15% trichloroacetic acid (TCA) to one of the groups of 300 μL aliquots, followed by incubation at −20 °C for 10 min. The supernatant, resulting from centrifugation at 1.000× *g* for 10 min at 4 °C, was then collected for carbohydrate measurement. The absorbance was read at 492 nm after samples and glucose standard concentrations were incubated for 30 min at room temperature by adding 200 µL of 5% phenol and 800 μL of H_2_SO_4_. The remaining pellet was resuspended by adding 500 μL of sodium hydroxide (NaOH) and heated to 60 °C for 30 min. For sample neutralisation, 280 µL of Hydrochloric acid (HCL) was used to finalise the samples. Total protein content was obtained following Bradford’s method adapted from BioRad’s Bradford micro-assay set up in a 96-well flat bottom plate [31], which uses bovine serum albumin for the calibration curve and a reading absorbance of 592 nm. Results of lipids, sugars, and proteins are expressed in mJ/mg organism. 

The last 300 µL aliquots were used to evaluate ETS activity. Following the method of De Coen and Janssen [29], 150 μL of homogenisation buffer (Tris base (0.3 M); Polyvinylpyrrolidone (0.45% (*w*/*v*)); 459 μM MgSO_4_; Triton X-100 (0.6% (*v*/*v*)) at a pH of 8.5) was added to the samples that were later centrifuged at 1000× *g* for 10 min at 4 °C. In the multi-well plate, 50 μL of the resulting supernatant was incubated with 150 μL of a buffered solution (Tris base (0.13 M) with Triton X-100 (0.27% [*v*/*v*]), 1.7 mM reduced nicotinamide adenine dinucleotide (NADH), and 274 μM reduced nicotinamide adenine dinucleotide phosphate (NADPH)) and 100 µL of INT solution (p-iodonitrotetrazolium; 8 mM). Absorbance was estimated kinetically at 490 nm over a 3 min period. Results are expressed in mJ/h/mg organism. 

All biomarker determinations were performed at 25 °C with the microplate reader MultiSkan Spectrum (Thermo Fisher Scientific, Waltham, MA, USA). 

### 2.9. Statistical Analysis

For analysing the results from the 28-day exposure bioassay, the emergence ratio (ER), the mortality rate (M), and the percentage of emerged individuals per day (ED) were calculated according to Savić-Zdravković et al. [32]. To analyse the differences between the control group and the various concentrations of PU-MPs, a one-way analysis of variance (ANOVA) was used, followed by Dunnett’s post hoc test. Results are presented as mean ± standard error of the mean (SEM). 

For the second bioassay, related to the short-term (of 10 days) exposure, and considering that there were only two groups, control vs. PU-MPs, the difference in the multiple parameters (larval body length, movement, and biochemical biomarkers), in *C. riparius* was evaluated by applying the unpaired two-tailed Student’s *t*-test. Results are presented as mean ± SEM. 

For all statistical tests, the significance level was set at *p* < 0.05. All data were analysed using the statistic software GraphPad Prism version 9.3.1 for Windows (GraphPad Software Inc., La Jolla, CA, USA). 

## 3. Results and Discussion

### 3.1. Effects on C. riparius Life History Traits after 28-d Exposure

**Figure 1:**
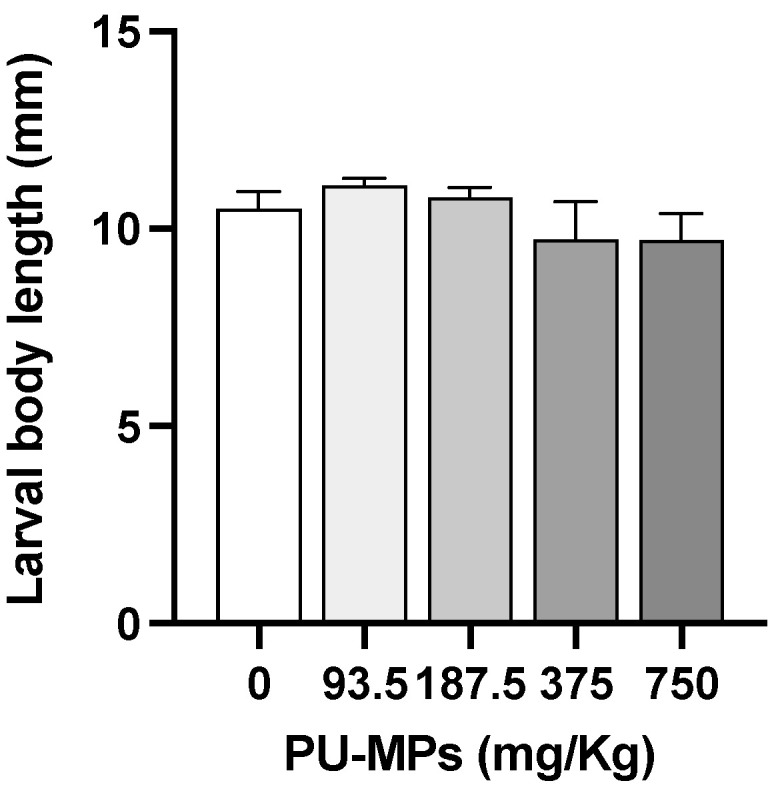
Effect of different concentrations of polyurethane microplastics (PU-MPs) on *Chironomus riparius* larval body length (n = 5). All values are presented as mean ± standard error of the mean. No significant differences between treatments were denoted.

The exposure to the various concentrations of PU-MP did not cause significant differences in larval body length in comparison with the control treatment, indicating that growth was similar in the presence or absence of PU-MPs when achieving the fourth instar (one-way ANOVA, F_(4, 20)_ = 1.190, *p* = 0.3454, Figure 1). The control group presented an average body length of 10.5 ± 0.4 mm (mean ± SEM). For the treatment groups, the average body length of larvae was 11.1 ± 0.2, 10.8 ± 0.3, 10 ± 1, and 9.7 ± 0.7 after exposure to 93.5, 187.5, 375, and 750 mg PU/Kg sediment, respectively. 

Our results contrast (to some extent) with previous investigations, where exposure to sediments contaminated with small-sized PE-MPs (32–64 µm) affected *C. riparius* larval body length (a proxy of growth), although at concentrations considerably higher and bigger sizes than the ones tested in this investigation [6]. The same species exposed to a mixture of polymers (PA, PE, PET, PS, PVC, and concentrations up to 80 g/cm^2^) revealed an increase in larval body mass and length [11]. Another chironomid, *C. tepperi*, exposed to PE-MP concentrations even smaller than those used in the present study (size ranges of 1–4, 10–27, 43–54, and 100–126 μm), also showed a reduction in larval body length [7]. Such differences in MPs effects on chironomids’ larval length are related not only to the number and size of incorporated MPs, but also to the polymer type, its potential additives, and organisms’ capacity to egest the particles to deal with physical and chemical hazardousness caused by MPs. 

Imagoes emergence passed the validity criteria, with an emergence ratio in controls of at least 70% [22]. There was no statistically significant difference in the emergence ratio (ER) or larvae mortality (M) between treatments (one-way ANOVA, F_(4, 20)_ = 1.400, *p* = 0.27; Table 2). Adults which emerged per day were also analysed and did not vary significantly, with an average of approximately three individuals per day (one-way ANOVA, F_(4, 20)_ = 1.190, *p* = 0.3454). In fact, the study carried out by Stanković et al. [11] obtained similar results, with no differences regarding larvae emergence and mortality ratio in *C. riparius* when exposed to a mixture of MPs (PET, PS, PVC, and PA, 10–400 µm, 8–80 g/m^2^ sediment). Notwithstanding, some deformities were observed in female imagoes wings.

**Figure 2:**
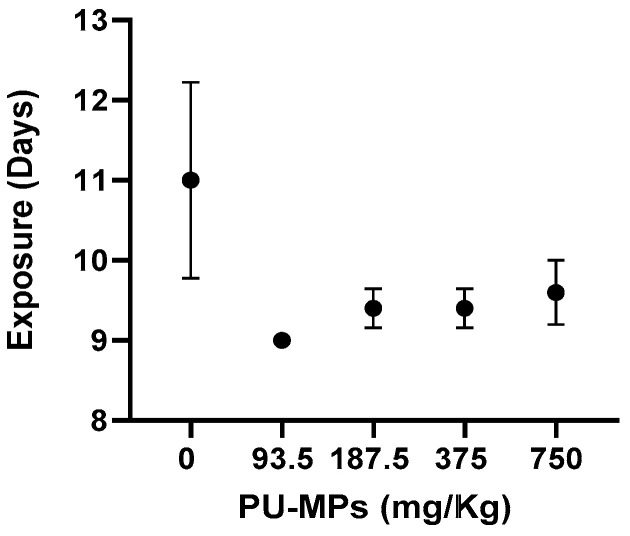
Effect of different concentrations of polyurethane microplastics (PU-MPs) on *Chironomus riparius* first day of emergence (n = 5). All values are presented as mean ± standard error of the mean. No significant differences between treatments were denoted.

The mean time until the first emergence can be observed in Figure 2. In the control group, the first emergence was noted after 11 ± 1 days of exposure, while in the presence of PU-contaminated sediment, the first emergence occurred after 9 ± 0 when exposed to 93.5 mg/Kg, 9.4 ± 0.2 days at 187.5 mg/Kg, 9.4 ± 0.2 days at 375 mg/Kg, and 9.6 ± 0.4 days at 750 mg/Kg. These results, although not significant (one-way ANOVA, F_(4, 20)_ = 1.663, *p* = 0.1980), showed anticipation in the first emergency of exposed larvae. These results contrast with previous studies, such as Silva et al. [6], who observed a delay in the emergence of females right from the first concentration tested (1.25 g/Kg sediment, which is considerably higher than the ones from this study), resulting in a 2–3-fold higher difference in the mean emergence time. The results on these organismal parameters suggest that PU-MP size and concentration did not seem to affect the reproduction (and eventual population dynamics) of *C. riparius* midges inhabiting contaminated freshwater sediments. However, it should be noted that the effects of environmental stressors such as MPs depend on features such as polymer size and dose. Using PE as a test polymer, Silva et al. [6] revealed that imagoes emergence was negatively affected by small-sized PE (32–63 μm) for concentrations higher than 1 g/kg, which contained a larger number of small particles. 

### 3.2. Presence of PU-MPs in Chironomus riparius Larval Digestive Tract, after 10 Days of Exposure

**Figure 3:**
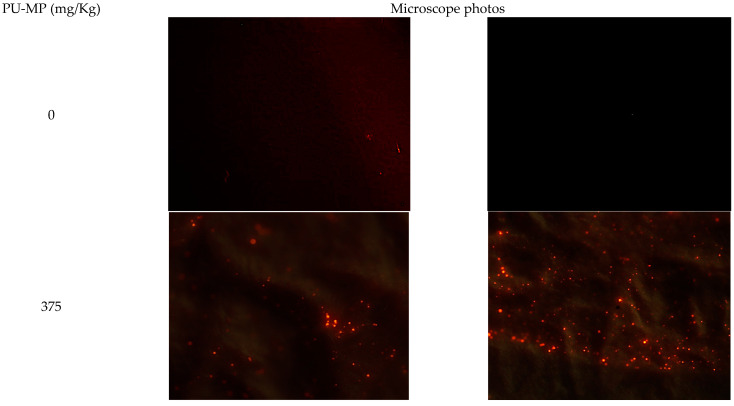
Polyurethane microplastics (PU-MPs) present in *C. riparius* four instar larvae digestive tract after a 10-day exposure to 0 (control condition, two random filters) and 375 mg PU-MP/kg of sediment (two random filters). Particles presenting red fluorescence are PU-MPs. Note: The presence of MPs in controls might be due to potential airborne contamination. Nonetheless, the number of particles was negligible compared to PU-MP treatments. Eyepiece magnification: ×10; Objective: ×40.

Chironomid larvae ingested PU-MP after 10 days of exposure to PU-MP NOEC (375 mg/kg), as several thousands of particles were observed in their gut (Figure 3). However, their quantification was compromised by a high number of particles and, due to microplastics’ physicochemical characteristics [33], high aggregation. Chironomids, particularly *C. riparius*, ingested and accumulated a considerably high number of PE-MPs of 32–63 μm (up to 2500 particles per organism) [6]. Scherer et al. [12] also reported the uptake of polystyrene (PS) beads of 1, 10, and 90 µm by *C. riparius* larvae, consuming 226 particles per hour. Another chironomid species, *Chironomus tepperi,* also incorporated PE-MPs with a strong tendency to agglomerate in the gut, which is visible using a stereo-microscope [7]. The smaller the microplastics’ size, the higher tendency to their accumulation (and translocation) within tissues [34,35]. Although no quantification is provided in this study, it is unquestionable the high amount present in their digestive tract that could (to some extent) induce sub-lethal effects, as discussed further. 

### 3.3. Effects on Larvae Behaviour and Homeostasis after 10-d Exposure

**Figure 4:**
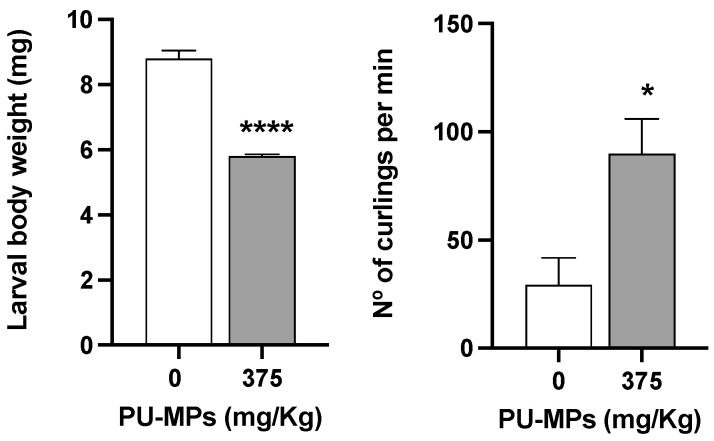
Effects of polyurethane microplastics (PU-MPs) in *Chironomus riparius* larval weight (left side; n = 5) and larvae locomotion (number of curlings and uncurlings per minute; right side; n = 4), after 10 days of exposure. All values are presented as mean ± standard error of the mean. *, **** denotes a significant difference from the control with *p* < 0.05 and *p* < 0.0001, respectively.

The exposure to 375 mg/kg of PU-MPs and its ingestion did not cause significant differences in larval body length compared to the control treatment (t = 0.2248, df = 34, *p* = 0.8235; Figure 4). The control group presented an average body length of 10.2 ± 0.2 mm (mean ± SEM), and the contaminated group had an average body length of 10.3 ± 0.2. This parameter remained around 10 mm for both treatments, which is not very different from Stanković et al. [11], which presented the same larval length in the control group. These results were expected since it is probably a reason for the outcome observed in larval emergence in the previous section, which proved not to be affected by PU-MPs. 

Despite the absence of effects on larvae length between contaminated and control larvae, the same is not observed for their weight and behaviour. As observed in Figure 4, *C. riparius* larval weight was significantly lower in contaminated larvae (34% less weight, t = 11.79, df = 8, *p* < 0.001). Conversely, contaminated larval presented significantly higher locomotion (t = 2.989, df = 6, *p* = 0.0244), with a mean difference of 60.53 curlings per minute between the contaminated and the control group (mean of the control group = 29.35; mean of contaminated group = 89.88). The increased locomotion in contaminated larvae could be related to their lower weight (as lighter weight in larvae, as in any other animal, is likely to ease their locomotion). Such greater activity from contaminated larvae can also be related to a potential increase in acetylcholinesterase activity (AchE, as a proxy of “organisms’ activity/movements” in macroinvertebrates) as observed in *C. riparius* larvae exposed to PE-MPs < 64 μm in size [9]. Authors attributed the increment in AchE to the peristaltic movements promoted by larvae as an attempt to egest microplastics retained in their gastrointestinal tract. This increase in larvae locomotion can, therefore, be reflected in energy expenditure.

**Figure 5:**
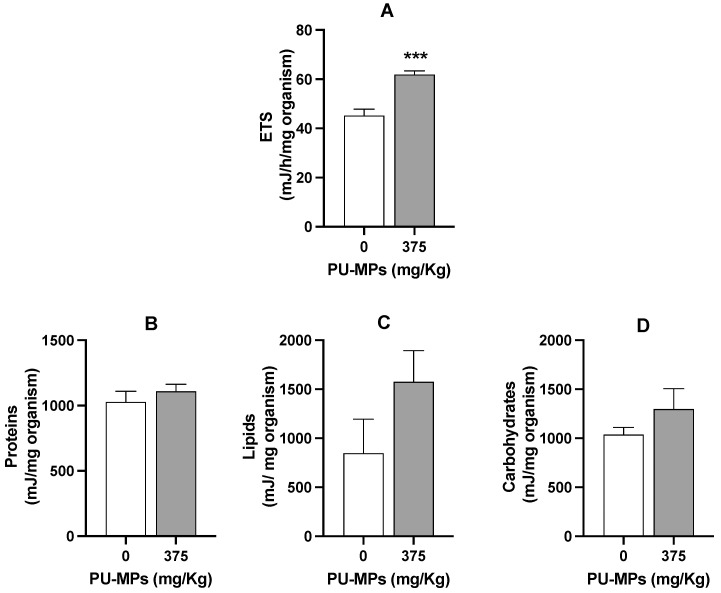
Effect of polyurethane microplastics (PU-MPs) on (**A**) aerobic energy production (electron transport system, ETS, mJ/h/mg organism), (**B**) proteins, (**C**) lipids, and (**D**) carbohydrates content (mJ/mg organism) of *Chironomus riparius* larvae after 10 days of exposure (n = 5). All values are presented as mean ± standard error of the mean. *** denotes a significant difference from the control (*p* < 0.001).

**Figure 6:**
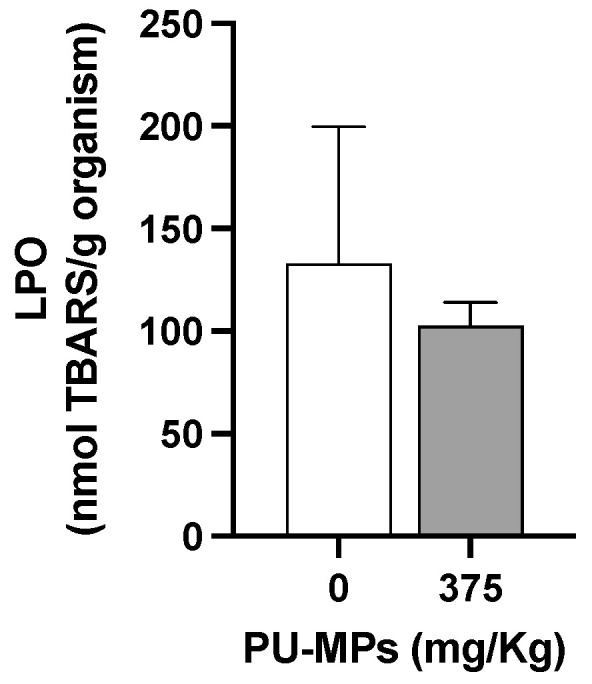
Lipid peroxidation (LPO) levels in *Chironomus riparius* larvae after 10 days of exposure to polyurethane microplastics (PU-MPs) (n = 5). All values are presented as mean ± standard error of the mean. No significant differences between treatments were denoted.

In fact, larvae exposed to PU-MPs revealed activation of aerobic energy production (in other words, one can infer in terms of activation on the metabolism, also reflecting an increase in energy expenditure) compared to the controls (Figure 5A). The fact that contaminated larvae were more active while foraging (curling/uncurling more) partially explains such energy consumption. However, despite the higher locomotion activity, including lower weight, contaminated larvae did not reveal a significant energy reserves shift, i.e., in terms of proteins (t = 0.8437, df = 8, and *p* = 0.4234, Figure 5B), lipids (t = 1.549, df = 8, and *p* = 0.1600, Figure 5C), and carbohydrates (t = 1.179, df = 8, and *p* = 0.2724, Figure 5D). A higher energy consumption (increased metabolism) can also be related to the activation of immune responses and the consequent trigger of antioxidant defences to fight reactive oxygen species (ROS). Silva et al. [20] used a different MP polymer (PE; 32–63 µm) and provided evidence of activation of the *C. riparius* immune system due to damage to the epithelial cells of the gut lumen. Effective activation of response mechanisms against ROS can be related to the absence of lipid peroxidation, as observed in Figure 6. Levels of LPO were not significantly altered with exposure to PU-MPs (t = 0.4500, df = 8, and *p* = 0.6647; Figure 6). Yet, to confirm such a hypothesis, an assessment of the antioxidant and detoxicant capacities of *C. riparius* larvae is required (e.g., assessment of catalase, glutathione peroxidase, glutathione-S-transferase activities, among others). Silva et al. [9] previously observed the expression of these antioxidant enzymes in *C. riparius* larvae, which registered an inhibition of CAT and GST activities after exposure to PE-MPs (particularly evident for larvae that ingested larger particles, 125–500 μm). A different response was observed in the annelid *Lumbriculus variegatus* after exposure to PE-MPs, which showed an increase in total glutathione in all size classes (PE-MPs; size-class A: 32–63, B: 63–125, C: 125–250, and D: 250–500 μm) and GST levels only on particles > 125 μm [26]. Conversely, CAT levels were not affected. This is evidence that the responses of these enzymes are highly dependent on the species and the organism’s capacity and mechanism efficiency to manage the uptake of MPs. 

Integrating all the results obtained in this study, *C. riparius* larvae exposed to 375 mg PU-MPs/kg could ingest a considerably higher number of PU-MPs. They were revealed to be lighter (but not smaller nor less nutritionally affected in terms of energy reserves) and more active when foraging, which was reflected in the activation of their aerobic metabolism. Notwithstanding, the behavioural and metabolic alteration did not originate quantifiable costs in terms of energy reserves, i.e., on protein, lipid, or sugar content on contaminated larvae, which may justify the absence of effects on larval growth and emergence. Therefore, the increased energy used for the locomotion and functioning of larvae was at the expense of energy allocated for the weight of the individuals. A long-term exposure involving a multigenerational assessment would bring intel on the potential (cumulative) sub-lethal effects of PU-MPs on *C. riparius* fitness.

According to Sokolova et al. [36], organisms facing stress allocate their energy for survival instead of storage, activity, or reproduction. However, it has been proved that the energy used is not proportionally deviated from those physiological processes. Indeed it has been shown that larvae of chironomids exposed to paraquat prefer to allocate energy for reproduction, whereas planarians decrease the reproductive outputs to ensure their survival [37]. In our particular case, it seems that chironomids use energy for their curling activity, probably trying to egest microplastics from inside their guts. Since the energy consumption increased and energy reserves were kept constant, there will be less energy available to increase their mass, but not their length, due to a decrease in cellular energy allocation (the ratio between the sum of the energy reserves and the energy consumption—ETS). Thus, partial life-cycle tests with chironomids determining the length and not using weight might underestimate some chronic effects of pollutants in growth-related parameters.

## 4. Conclusions

This study showed that exposure to environmentally relevant concentrations (up to 750 mg/kg) of PU-MPs (a thermoset plastic) with a size between 7.0 and 9.0 μm seemed to induce sub-lethal effects on larvae of the freshwater dipteran *Chironomus riparius*, considering their decreased weight and abnormal high curling/uncurling rate. These effects are clear signs of toxicity at the organism level. Nevertheless, contaminated larvae were revealed to be lighter (but not smaller) and more active when foraging, which was reflected in the activation of their aerobic metabolism. Notwithstanding, no alteration in energy reserves (proteins, carbohydrates, or lipids) was observed, which may justify the absence of effects on larval length and emergence. Thus, it seems that larvae ingesting PU-MPs compromise the energy usually allocated for their weight, but not to their length, which allows completion of the four larval stages that are needed to emerge as adults. However, long-term exposure involving multigeneration would bring intel on the potential (cumulative) sub-lethal effects of PU-MPs on *C. riparius* fitness and the success of adults in reproduction. 

This investigation also brings new evidence on the behavioural effects induced by small-sized microplastics that have been neglected so far. Despite the lack of significant effects on larval length and emergence, the change in their behaviour can, for instance, increase their predation by ferocious organisms that target very active prey (e.g., planarians and fish). Such behavioural alterations might also trigger the release of chemical clues (kairomones) enabling the detection by predators [38]. Thus, behaviour alterations can impact not only individual fitness, but also population dynamics, species interactions, and ecosystem functions [39].

Microplastics (thermoplastics or thermosets) proved to have effects on chironomids’ behaviour, life-history traits, and/or biochemical and cellular response mechanisms. Even though most alterations have been reported for concentrations not yet reported in the field, it is undeniable that if no mitigation or remediation actions are in place, they will likely be achieved in the near future. Chironomids, along with other benthic macroinvertebrates, underpin the delivery of many ecosystem services and functions in freshwaters, such as nutrient cycling and water quality. Thus, the ecological risk assessment of microplastics in freshwater is crucial to successfully implementing mitigation strategies to ensure clean water provisioning and to prevent the loss of freshwater biodiversity. 

## Figures and Tables

**Table 2:** Effect of different concentrations of polyurethane microplastics (PU-MPs) on *Chironomus riparius* life cycle endpoints. Emergence ratio (ER)-% of emerged individuals at the end of the experiment; Mortality rate (M)-% of dead individuals at the end of the experiment; and average % of emerged individuals per day (ED). All values are presented as mean ± standard error of the mean. No significant differences between treatments were denoted.

Parameters	PU-MPs (mg/kg)				
	0	93.5	187.5	375	750
Emergence ratio (ER) ± SEM	84 ± 7	92 ± 5	84 ± 7	100 ± 0	92 ± 5
Mortality (M) ± SEM	16 ± 7	8 ± 5	16 ± 7	0 ± 0	8 ± 5
Emerged individuals/day (ED) ± SEM	2.3 ± 0.9	3 ± 1	2.3 ± 0.9	2.8 ± 0.9	2.6 ± 0.6

## Data Availability

Not applicable.
